# Post-Partum Depression Lactating Rat Model for Evaluating Ketamine’s Safety as a Pharmacotherapeutic Treatment: Roles in Cardiac and Urinary Function

**DOI:** 10.3390/jcdd9090299

**Published:** 2022-09-07

**Authors:** André Rinaldi Fukushima, Pedro Enrique Navas-Suárez, Juliana Weckx Peña Muñoz, Esther Lopes Ricci, Luís Antônio Baffile Leoni, Érico C. Caperuto, Leandro Yanase, Jeferson Santana, Elias de França, Jan Carlo Morais O. Bertassoni Delorenzi, Alcides Felix Terrivel, Gláucio M. Ferreira, Mario Hiroyuki Hirata, Lorena de Paula Pantaleon, Julia Zacarelli-Magalhães, Gabriel Ramos de Abreu, Paula A. Faria Waziry, Maria Aparecida Nicoletti, Helenice de Souza Spinosa

**Affiliations:** 1 Faculdade de Medicina Veterinária e Zootecnia, Universidade de São Paulo, São Paulo 05508-270, SP, Brazil; 2 Faculdade de Ciências da Saúde IGESP (FASIG), São Paulo 01301-000, SP, Brazil; 3 Centro de Treinamento Veterinário, São Paulo 01532-000, SP, Brazil; 4 Centro de Ciências Biológicas e da Saúde Universidade Presbiteriana Mackenzie, São Paulo 01302-907, SP, Brazil; 5 Grupo de Estudos e Pesquisa Aplicada em Metabolismo do Exercício, São Paulo 86039-100, SP, Brazil; 6 Department of Clinical and Toxicological Analyses, School of Pharmaceutical Sciences, University of Sao Paulo, São Paulo 05508-000, SP, Brazil; 7 Western Atlantic University School of Medicine (WAUSM), Plantation, FL 33324, USA; 8 School of Pharmaceutical Sciences, University of Sao Paulo, São Paulo 05508-000, SP, Brazil

**Keywords:** cardio-chemical analysis, neurohormones, cardiotoxicity, postpartum depression, animal model

## Abstract

Depression is one of the world’s most common and mentally disabling illnesses. Post-partum depression is a subtype of depression that affects one in seven women worldwide. Successful pharmacological treatment must consider the consequences for both, since the mother–child bond is fundamental for the well-being of both mother and infant as well as the general development of the newborn. Changes in maternal physiology and/or behavior can significantly influence the development of breastfed infants. Ketamine has been extensively studied for use as an antidepressant due to its mixed mechanisms of action. Safety and efficacy studies in the cardiovascular and urinary systems of a lactating postpartum depression animal model are essential for contributing toward ketamine’s clinical use in the respective patient population. Thus, this project aimed to study the implications of postpartum maternal exposure to ketamine during lactation on the cardiovascular system of female rats submitted to the depression induction model by maternal separation. This model promotes depressive effects through stress caused by the interruption of mother–infant bond early in the offspring’s life. To achieve depression, each dam was separated from her offspring for 3 h per day, from post-natal day 2 (PND2) to PND12. Experimental groups received daily treatment with either 5, 10, or 20 mg/kg of ketamine intraperitoneally during the lactation period, from PND2 to PND21. Behavioral tests consisted of the maternal and aggressive maternal behavior tests, the olfactory preference test, and the forced swim test. A technique for the detection of catecholamines and indoleamines in the heart muscle was developed for the experimental model groups. The histopathological evaluation was performed on these animals’ cardiac muscles and urinary bladders. Our findings suggest that ketamine is safe for use in postpartum depression and does not induce cardiovascular and/or urinary systems toxicity.

## 1. Introduction

Postpartum depression (PPD), also known as postnatal depression (PNATD), is a major depressive episode and the most common complication of childbearing that affects up to 20% of mothers during the first 3 months after delivery [1]. PPD displays a unique challenge due to unavoidable psychological, social, and biological changes. The latter includes both hormonal and immunological shifts that are solely experienced during postpartum [2], which could greatly benefit from pharmacotherapeutics. Despite its high occurrence, PPD remains underdiagnosed and undertreated. Untreated PPD has short-term and long-term detrimental consequences for mothers and neonates. Furthermore, untreated PPD is the primary cause of maternal suicide during the first postpartum year [3]. Despite the alarming consequences of PPD, many afflicted mothers do not seek psychotropic drugs while breastfeeding due to concerns over adverse effects on their bodies and on neonates. Furthermore, the available pharmacotherapeutics for major depressive disorders (MDD) are not effective due to the fact that approximately 30% of patients are drug-resistant or non-responsive [4]. Ketamine, a phencyclidine derivative, is an antagonist of the N-methyl-D-aspartate receptor (NMDAR), one of the newest approved treatments for MDD. Ketamine has a rapid antidepressant and anti-suicidal effect (12–24 h) when compared to conventional drugs that take up to 4 weeks post-initiation of treatment to start showing effects [5,6].

### 1.1. General Mechanism of Action and Safety of Ketamine

Ketamine’s antidepressant mechanism is still not fully understood. Antidepressant as well as dissociative effects may be influenced by dose, frequency, route of administration and sex differences that are largely dependent on hormonal levels [7].

Studies have indicated that ketamine exerts effects on several types of receptors in the nervous system, including the inhibition of presynaptic and postsynaptic NMDARs in gamma-aminobutyric acidergic (GABAergic) interneurons. The mechanism also activates postsynaptic a-amino-3-hydroxy-5-methyl-4 isoxazole propionic acid receptors (AMPARs), the brain-derived neurotrophic factor tyrosine receptor kinase B (BDNF-TrkB) signaling pathway, opioids, and monoamines receptors, as well as on voltage-gated calcium channels [8,9,10]. The main interaction of ketamine occurs with the NMDA glutamatergic receptor, which is coupled to an ion channel that has excitatory properties [9]. Ketamine is a non-competitive calcium channel antagonist: Once the drug is bound to the receptor, it blocks calcium influx, which prevents cell depolarization [9,11].

Ketamine has been safely used as a short-term dissociative anesthetic since 1970; however, the direct impact from long-term ketamine treatment on cognition or on other organ systems such as cardiovascular and genitourinary is not currently known. Importantly, there have been no long-term controlled trials for ketamine that directly investigated such outcomes, and further studies are necessary [12].

### 1.2. Effects on Cardiac and Urinary Function

The action of ketamine on non-NMDA glutamatergic receptors is most likely mediated by the glutamate/nitric oxide/cGMP system. Signal transduction via non-NMDA receptors stimulates nitric oxide production, which increases cGMP’s concentration. Ketamine inhibits non-NMDA receptors and, consequently, inhibits the action of the enzyme nitric oxide synthase, halting nitric oxide production [13,14]. Inhibition of nitric oxide synthase seems to play an essential role in the analgesic property of ketamine, in addition to possibly augmenting neuroprotection and activating the parasympathetic system [8].

Considering the cardiovascular system, nitric oxide synthase inhibition can lead to cardiotoxicity, which is associated with coronary vasoconstriction. Consequently, this leads to an increase in the risk of acute ischemic myocardial infarction [15].

Ketamine can increase right ventricular workload and pulmonary artery pressure, causing pulmonary hypertension [16]. Following the same rationale, when ketamine is used as an anesthetic, it can cause episodes of tachycardia and hypertension, which are considered complications that can compromise myocardial functions [17].

The toxic effects of ketamine on cardiac arteries result from sympathomimetic activity, which promotes the loss of cardiovascular protection [18,19,20]. A previous study employing a rat animal model had observed that increasing the administered dose of ketamine promoted an increase in endogenous serum catecholamines (adrenaline, noradrenaline, and dopamine) [21]. The elevated levels of catecholamines promoted binding to specific receptors in the heart and blood vessels that resulted in potentiated positive chronotropic and inotropic effects. Consequently, such stimuli caused the heart muscle to increase oxygen consumption and simultaneously reduce diastolic blood pressure and coronary perfusion, which can lead to severe tissue damage [18].

Li et al. showed that a single dose of ketamine can cause subtle cardiovascular changes, which are attributed to oxidative stress in cardiac tissue [22]. The group has also shown that prolonged use of ketamine could cause severe oxidative stress, and that cardiotoxicity was directly proportional to the duration of treatment [18].

Banerjee et al. suggested that the pathogenesis of myocardial lesions can be classified as multifactorial since it can be activated by two distinct pathways: the catecholaminergic pathway and the nitric oxide synthase pathway, which are both capable of generating oxidative stress [23].

The adverse cardiovascular outcome is not the only observed unfavorable effect of ketamine. The literature also describes that ketamine can cause uropathy based on data collected from clinical, biochemical, and histological studies from patients, as well as from studies carried out using animal models [24]. The bladder tissue, or urothelium, can also be affected by ketamine use. Several studies have confirmed that ketamine or its metabolites interact directly with the urothelium, resulting in a defective bladder epithelial barrier, thus causing the development of ketamine-induced cystitis [25,26,27]. However, little is known about the pathogenesis or mechanisms of ketamine in the urinary system [24].

Considering that the clinical use of ketamine has expanded from anesthetic to antidepressant, it is feasible to consider it for treating postpartum depression. However, there is little information for proper dosage or route of administration for the latter use. Furthermore, due to the unique physiological and immunological changes that occur after pregnancy, it is crucial to determine whether adverse effects of ketamine are exacerbated during that stage. Here we employed the rat animal model of postpartum depression and evaluated the effects of ketamine exposure during lactation on maternal behavior outcome and on the cardiovascular system and urinary bladder. Behavior outcome was accessed by the maternal behavior test, the aggressive maternal behavior test, the olfactory preference test, and the forced swim test. We developed a novel technique for detecting and quantifying neurohormones in cardiac tissue, which may prove valuable for determining markers of heart disease progression. No significant histopathological differences were observed between the groups evaluated, indicating that ketamine is a safe pharmacological treatment for postpartum depression during lactation.

## 2. Materials and Methods

### 2.1. Animals

The first pregnancy of approximately 90-day-old female rats (Rattus norvegicus) was employed for the current studies. Dams and their offspring were obtained from the vivarium of the Institute of Biomedical Sciences of the University of São Paulo. The animals were housed in polypropylene cages (41 × 34 × 18 cm) with autoclaved wood shavings as bedding and kept in a room with a relative humidity of 55% ± 10, temperature of 22 °C ± 2, and a 12-h cycle of light and dark (light on at 6 a.m. and off at 6 p.m.). Food and water were provided ad libitum throughout the experimental procedure.

The experiments were conducted at three facilities: (i) the Faculty of Veterinary Medicine and Animal Science at the University of São Paulo (FMVZ/USP), (ii) the Mackenzie Presbyterian University (UPM), and (iii) the University of Chicago. The experimental protocol was approved by the following institutions: (a) the FMVZ’s Committees on Ethics for the Use of Animals (CEUA)/USP, under protocol number 2099010218, and (b) by the CEUA of Universidade Presbiteriana Mackenzie (UPM) under protocol number 168-04-2018.

### 2.2. Experimental Design

Seventy lactating adult rats were divided into 7 groups A through G (n = 10 females/group), according to Table 1. Females were induced to postpartum depression through the maternal separation model from postnatal day 2 (PND2) to PND12. Treatment with different doses of ketamine (groups C, D, E, and F) or saline solution (group B) was performed intraperitoneally during the lactation period, from PND2 to PND21, except for group G, which received a single dose of ketamine 10 mg/kg in PND12 as described in Table 1.

### 2.3. Model of Depression Induction by Maternal Separation

The maternal separation model was used to induce postpartum depression in rats, as described by Vetulani et al. [28]. This model promotes depressive effects through stress caused by the interruption of the mother–newborn bond. Induction of depression was achieved by separating each dam from her offspring for 3 h a day, from PND2 to PND12, from 10 am to 1 pm. The pups were removed from the mothers’ home cages and placed in small plastic boxes (18 × 13 × 11 cm) containing bedding from the mothers’ home cages. These small boxes were then placed on a thermal blanket with a temperature of 32 °C ± 2 and kept in the same room where the mothers were housed. This setting allowed the mothers to hear and smell their pups but did not allow physical or visual contact with them.

### 2.4. Timeline and Experimental Design

**Figure 1:**
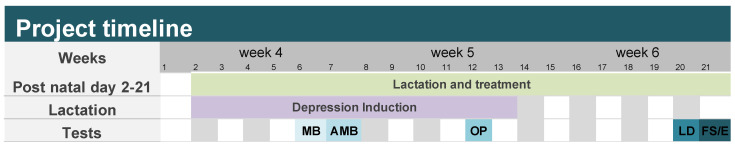
Timeline of experimental procedures. MB: maternal behavior; AMB: aggressive maternal behavior; OP: olfactory preference; LD: light/dark box; FS/E: forced swim test and euthanasia.

Pregnant rats were kept undisturbed during the gestational period of 21 days (weeks 1–3). Figure 1 summarizes the timeline of procedures, treatments, and behavioral tests. Briefly, the litters were born on Day 1 of Week 4. Induction of PPD occurred between PND2 and PND13. Lactation and treatment took place between PND2 and PND22. Behavioral tests were used to verify if the depression model was effective for the rats submitted to the model. In PND5, the maternal behavior test (MB) was performed, and in PND6, the aggressive maternal behavior test (AMB) was performed. In PND12, the olfactory preference test (OP) was performed, and in PND20, the light/dark box test (LD). In PND21, the offspring were weaned and on the same day the mothers were submitted to the forced swimming test (FS), then euthanized (E) by decapitation for collection of cardiac muscle and bladder for histopathological and cardiochemical analyses.

### 2.5. Histopathological Evaluation

For histopathological evaluation, hearts and urinary bladders were fixed in 10% buffered formalin and processed to make histological slides. For the anatomopathological study of the heart, the following stains were used: hematoxylin-eosin (HE—differentiates basophilic parts by hematoxylin, and acidophilic parts by eosin); Masson’s trichrome (distinguishes cells of connective tissue origin); and Mallory’s phosphotungstic acid hematoxylin (PTAH)—distinguishes striae of muscle fibers [blue-black], from collagen [red-orange]). Urinary bladders were processed to prepare histological slides using HE staining.

### 2.6. Cardiochemical Assessment

To study the cardiotoxic and structural effects of exposure of dams to different doses of ketamine (Groups: D: ketamine 5 mg/kg; E: ketamine 10 mg/kg; F: ketamine 20 mg/kg and G: single dose of Ketamine 10 mg/kg) during lactation, 60 lactating dams were divided into 6 groups (n = 10 females/group). The dams were euthanized at PND21. Immediately following euthanasia, their hearts and urinary bladders were collected for analysis.

The collected hearts were placed on ice to preserve the organ for dissection and examination of structure. The cardiac apex was placed in a microtube and conditioned in liquid nitrogen for immediate freezing. Then, samples were weighed and stored at −80 °C for processing within 30 days.

Processing of the apex took place by homogenization in 0.1 M perchloric acid (CIHO_4_) containing 3,4-dihydroxybenzylamine acid (DHBA—internal standard). The volume of acid used was 10 times the apex volume, and homogenization was achieved using a high frequency sonicator pen.

After homogenization, the structures were kept at 10 °C overnight for precipitation of proteins and nucleic acids. The following day, the samples were centrifuged at 10,000 rpm (Eppendorf^®^-5804 R centrifuge) at 4 °C for 30 min. The supernatant was removed and placed in microtubes for storage at −80 °C for subsequent analytical quantification of neurotransmitters and metabolites.

Analyzes were done by high-performance liquid chromatography with electrochemical detection (HPLC-ED) at the Laboratory of Applied Pharmacology and Toxicology at FMVZ/USP. The Shimadzu^®^ Model 20A chromatograph was used, consisting of an automatic injector with a variable volume injection valve (between 1 and 100 µL), quaternary flow pumps, a chromatographic column (150 × 4.6 mm, Shimpak^®^—ODS C 18) with power strip, and an Antec^®^ Decade^®^ electrochemical detector. The technique of reserve phase chromatography with ion pairing (heptanesulfonic acid) was used, which is based on partition or absorption chromatography. For the analyses, a temperature of 60 °C and timing up to 24 min were used to obtain representative peaks.

The mobile phase used was an isocratic system formed by 0.02 M citrate buffer, 5% acetonitrile, 0.12 nM sodium ethylenediamine tetra-acetic acid (EDTA), and 0.0556% 1-heptanesulfonic acid (has). The pH was adjusted to 2.71 with orthophosphoric acid (H_3_PO_4_). The mobile phase was filtered in a vacuum system before being applied to the HPLC-ED. After this procedure, the column was equilibrated by circulation in the overnight system. The column was packed at a flow rate of 1.2 mL/min. The detector was held at a potential of 0.8 V at the working electrode.

Standard solutions of monoamines were prepared using a concentration of 1 nM of each neurotransmitter or its metabolites diluted in 0.1 M hydrochloric acid containing 0.02% sodium metabisulfite. The standards were stored at −80 °C. For analysis, the standards were thawed and diluted from 1:2500 to 1:10,000 in 0.1 M perchloric acid. At least two standards were daily injected into the HPLC-ED for reference: once before and once after injection of experimental dosages. The equipment was standardized daily, once at the beginning and once at the end of the experimental analysis, with a working solution of known concentrations of neurotransmitters, their metabolites and DHBA, diluted in perchloric acid, 0.1M, containing EDTA and sodium metabisulfite.

The limit of detection was 0.25 and 1 ng for all analytes. The limit of quantification was 10 ng/g of cardiac tissue or 1.0 ng per 60 ng of supernatants, and the recovery rate was over 80%.

Quantification was performed for:

- Adrenaline (ADR);
- Noradrenaline (NOR) and its metabolite vanillylmandelic acid (VMA);
- Dopamine (DA) and its metabolites, 4,4-dihydroxyphenylacetic acid (DOPAC) and homovanillic acid (HVA);
- Serotonin (5HT) and its metabolite 5-hydroindole, 3-acetic acid (5HIAA).

The turnover (renewal rate) was also calculated through the metabolite/neurotransmitter ratio of NOR, DA, and 5HT as follows: VMA/NOR, DOPAC/DA, HVA/DA, and 5HIAA/5-HT.

### 2.7. Statistical Analyses

Neurotransmitter dosage data were analyzed using GraphPad Prism software. Bartlett’s test was used to verify the homoscedasticity of the data. When the data were parametric, one-way ANOVA was used, followed by Dunnett’s test, when there was only one factor to be evaluated. If there were more than one factor to be evaluated, two-way ANOVA with repeated measures was used, followed by the Bonferroni post-test. Results were expressed as mean ± standard error or median and their respective limits. Differences between groups were considered statistically significant at * *p* < 0.05, ** *p* < 0.01, and *** *p* < 0.001.

## 3. Results

The histological anatomopathological evaluation of the cardiac tissue of the rats submitted to induction of postpartum depression by maternal separation and treated with different doses of ketamine during lactation did not show any noteworthy alterations between the groups, in none of the three stains examined (HE, Masson’s trichrome, and PTAH).

**Figure 2:**
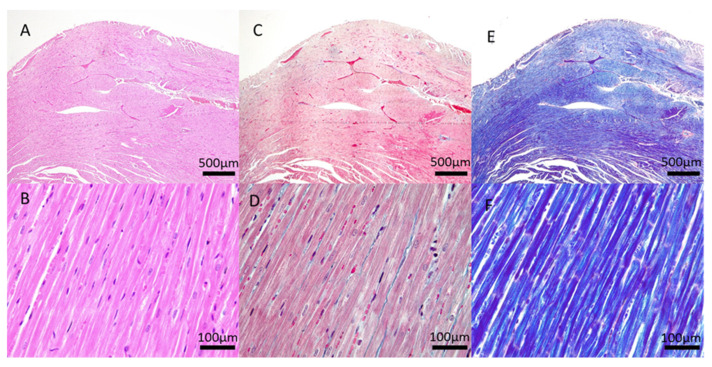
Histological photomicrographs of the cardiac tissue of rats in the control group show normal patterns of staining (**A**) HE—500 μm, (**B**) HE—100 μm, (**C**) Masson’s trichrome, (**D**) Masson’s trichrome—100 μm, (**E**) PTAH—500 μm and (**F**) PTAH—100 μm.

**Figure 3:**
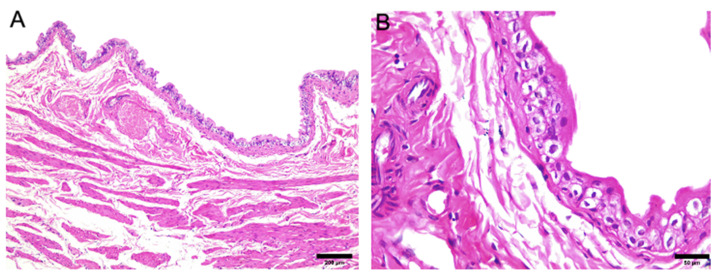
Histological photomicrographs of the urinary bladder tissue of rats in the control group show normal patterns of staining (hematoxylin-eosin staining)—(**A**) HE—200 μm and (**B**) HE—20 μm.

Figure 2 and Figure 3 illustrate the cardiac and urinary bladder tissue of a dam in the control group. The tissue was submitted to the three different stains mentioned above.

**Figure 4:**
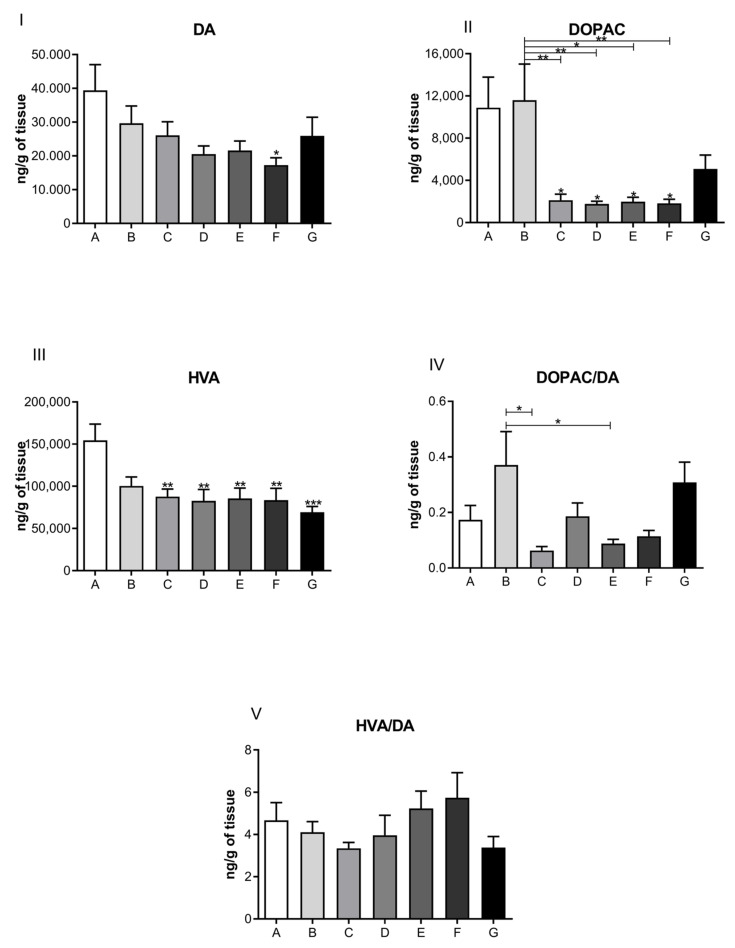
Neurotransmitter levels related to the dopaminergic system in the cardiac muscle of rats submitted to the postpartum separation depression model, treated or not with different doses of ketamine intraperitoneally. Panel (**I**): Dopamine levels (ng/g); Panel (**II**): 4,4-dihydroxyphenylacetic acid (DOPAC) levels (ng/g); Panel (**III**): homovanillic acid (HVA) levels (ng/g); Panel (**IV**): ratio of metabolite DOPAC/DA (ng/g); Panel (**V**): ratio of metabolite HVA/DA (ng/g). A—control group (normal, not treated); B—control and treated with saline; C—depressed and treated with saline; D—depressed + ketamine 5 mg/kg; E—depressed + ketamine 10 mg/kg; F—depressed + ketamine 20 mg/kg; G—depressed + single dose of ketamine 10 mg/kg group. The means and the respective standard errors are presented. N = 10. Animals per group * *p* < 0.05, ** *p* < 0.01, and *** *p*< 0.001 one-way ANOVA followed by Bonferroni test.

**Figure 5:**
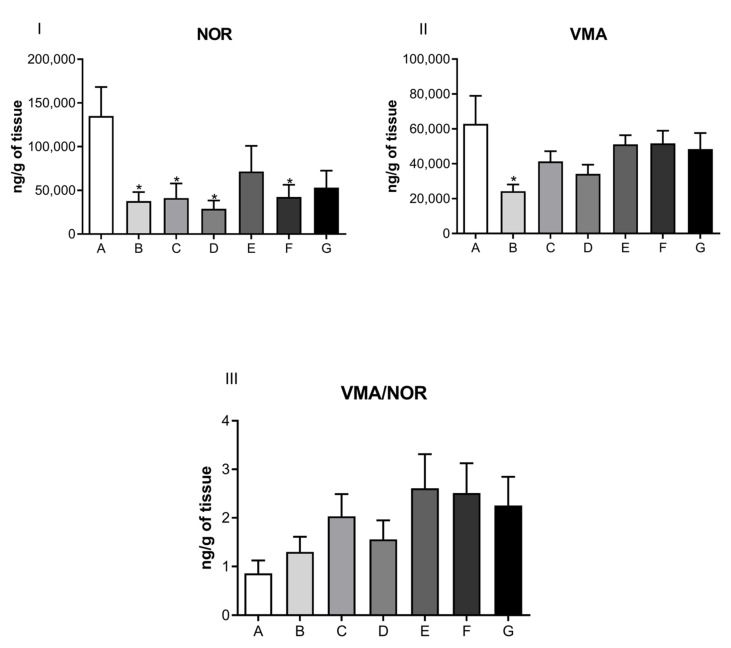
Neurotransmitter levels related to the noradrenergic system in the cardiac muscle of rats submitted to the depression model, treated or not with different doses of ketamine intraperitoneally. Panel (**I**): noradrenaline (NOR) levels (ng/g); Panel (**II**): vanillylmandelic acid (VMA) levels (ng/g); Panel (**III**): ratio of VMA/NOR (ng/g). A—control group (normal, not treated); B—control and treated with saline; C—depressed and treated with saline; D—depressed + ketamine 5 mg/kg; E—depressed + ketamine 10 mg/kg; F—depressed + ketamine 20 mg/kg; G—depressed + single dose of ketamine 10 mg/kg group The means and the respective standard errors are presented. N = 10. Animals per group * *p* < 0.05, ** *p* < 0.01 and *** *p* < 0.001 one-way ANOVA followed by Bonferroni test.

**Figure 6:**
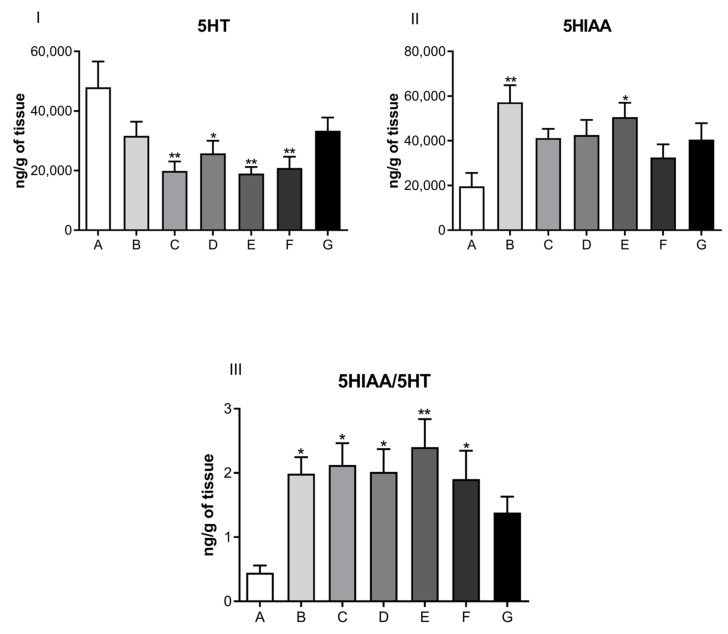
Neurotransmitter levels related to the serotonergic system in the cardiac muscle of dams submitted to the postpartum depression model, treated or not with different doses of ketamine intraperitoneally. Panel (**I**): serotonin (5HT) levels (ng/g); Panel (**II**): serotonin metabolite 5-hydroindole, 3-acetic acid (5HIAA) levels (ng/g); Panel (**III**): ratio of 5HIAA/5-HT (ng/g). A—control group (normal, not treated); B—control and treated with saline; C—depressed and treated with saline; D—depressed + ketamine 5 mg/kg; E—depressed + ketamine 10 mg/kg; F—depressed + ketamine 20 mg/kg; G—depressed + single dose of ketamine 10 mg/kg group. The means and the respective standard errors are presented. N = 10. Animals per group * *p* < 0.05, ** *p*< 0.01, and *** *p*< 0.001 one-way ANOVA followed by Bonferroni test.

**Figure 7:**
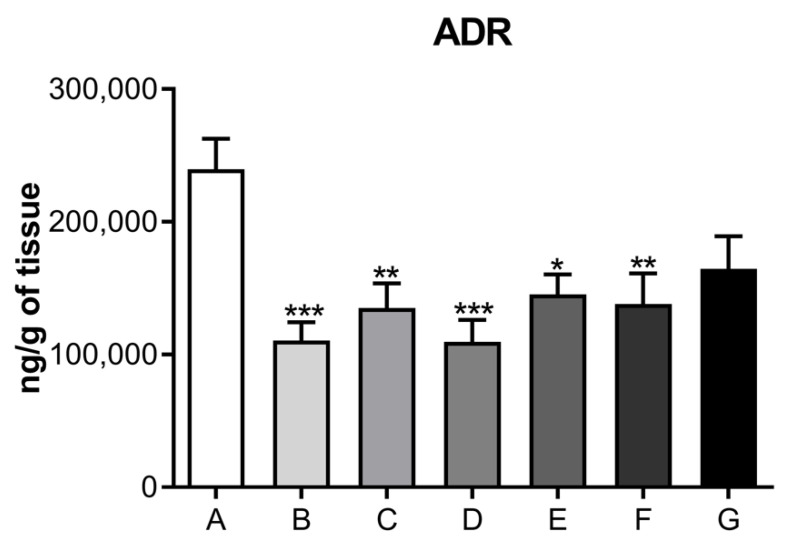
Level of neurotransmitters related to adrenaline in the cardiac muscle of dams submitted to the postpartum depression model, treated or not with different doses of ketamine intraperitoneally. The means and the respective standard errors are presented. N = 10. Animals per group * *p* < 0.05, ** *p* < 0.01 and *** *p* < 0.001 one-way ANOVA followed by Bonferroni test. A—control group (normal, not treated); B—control and treated with saline; C—depressed and treated with saline; D—depressed + ketamine 5 mg/kg; E—depressed + ketamine 10 mg/kg; F—depressed + ketamine 20 mg/kg; G—depressed + single dose of ketamine 10 mg/kg group.

The results from the direct measurement of neurohormones in cardiac muscle, performed by a high-performance liquid chromatography coupled to an electrochemical detector, are presented in Figure 4, Figure 5, Figure 6 and Figure 7 in the form of bar graphs. The statistical test used for comparison between groups was one-way ANOVA followed by the Bonferroni test and was considered as significant when the difference per group was *p* < 0.05.

Figure 4 illustrates a comparison of the levels of dopaminergic neurotransmitters obtained from cardiac muscles of postpartum depression dams that were treated (or not) with ketamine at the specified dosages and timelines. Notice that the levels of dopamine (panel I) for both control groups (A, B, and C) and the one-dose treatment group (G) did not show significant deviations from control. Groups D, E, and F show a decreasing trend in dopamine levels. However, only the group that received the highest repeated ketamine dose of 20 mg/kg (group F) showed a statistically significant decrease in dopamine (*p* < 0.05). Levels of DA metabolites DOPAC and HVA were significantly reduced for all groups treated with ketamine, having p values of 0.001 and 0.05 (Figure 4, panels II and III, groups C–G). Panels IV and V show a ratio of metabolites in comparison to DA.

Neurotransmitter levels related to the noradrenergic system are shown in Figure 5. No significant difference was observed for NOR (Panel I), its metabolite, VMA (Panel II), or the ratio of VMA/NOR (Panel III) in the cardiac muscle of rats submitted to the depression model, among control and treated groups. Subsequently, Figure 6 illustrates the levels of serotonergic system neurotransmitters, and Figure 7 displays ADR levels corresponding to the different animal groups, respectively.

## 4. Discussion

PPD is both clinically and experimentally understudied as well as underdiagnosed. Available pharmacotherapy for PPD coincides with drugs used for major depressive disorder. Ketamine has been shown to treat depression and suicidality effectively [29], and the drug is a fast-acting antidepressant that takes effect after a single dose. Ketamine’s effects can last for weeks or months [29,30], minimizing potential risks associated with continuous intake. Interestingly, new evidence suggests that its metabolites might have antidepressant properties, which may help explain the prolonged antidepressant effects of ketamine [31].

Ketamine has been studied for over 50 years; however, concerns over adverse effects remain and further research is much needed to establish drug safety and broaden the patient population for inclusion of postpartum lactating mothers. Risk factors of frequent ketamine use for the general population include cardiovascular and urothelium ulcerative cystitis [32,33].

In the literature, ketamine studies have employed doses and exposure durations that are related to three scenarios: (a) the use of ketamine as a drug of abuse for recreational purposes, associated or not with ethanol [34]; (b) the evaluation of ketamine as a general anesthetic, used in doses and exposure consistent with the anesthetic-surgical practice [35]; and (c) the clinical use of ketamine as an antidepressant [29].

Our studies expand the clinical antidepressant use of ketamine to include the treatment of postpartum depression, considering the systemic vulnerability of such patient population. It is crucial to investigate whether short-term use of ketamine might cause exacerbated effects that are not observed in the general population. Moreover, since safety studies are lacking on the use of ketamine for PPD, practitioners are currently instructed to avoid ketamine treatment in lactating mothers [29].

Here we have used the induced PPD rat model to evaluate possible detrimental effects that ketamine might exert on the cardiovascular and urinary systems. The doses and duration of treatment followed the established protocols that have shown effectiveness and safety for the general population [29]. Taking duration and dosage into consideration, our studies demonstrated that ketamine is safe for the proposed posology for PPD treatment, which contradicts results described by Wang and Yeung [36,37]. Wang and Yeung experimental design focused on an animal model that investigated long-term ketamine use as a recreational drug in combination with ethanol and showed central nervous system (CNS) toxicity, urinary system toxicity, and cardiotoxicity. Such discrepancies are most likely due to different doses of ketamine, different duration of treatment, and patterns of use [37]. Their studies concluded that the association of ketamine with alcohol turns is highly cardiotoxic [38].

Li et al. [22] conducted a comparable animal models (rats and rabbits) study to Wang and Yeung, which focused on the use of ketamine as a recreational drug [22]. Additionally, Li et al. evaluated the effects of ketamine and metoprolol (β-blocker used to treat angina and heart failure) on cardiac function, electrophysiological disorders, cardiac collagen, cardiomyocyte apoptosis, and proteins related to remodeling [22]. In rabbits treated with ketamine alone, there was a decrease in left ventricular ejection fraction, a decrease in ventricular conduction velocity, and an increase in susceptibility to ventricular arrhythmia. In ketamine-alone-treated rats, the cardiac collagen volume fraction and the number of apoptotic cells were higher than in controls. Furthermore, recreational, or chronic ketamine treatment increased sympathetic activation as well as levels of inflammatory markers. Noteworthily, ketamine increased the production of catecholamines, which can cause oxidative damage to the heart. The present study, which focused on the short-term use of ketamine, did not show significant lesions in the cardiac tissue of treated animals compared to the control group (Figure 2).

A thorough drug evaluation requires detailed analyses of neurotransmitters, which is important for general toxicology, pharmacology, and physiology. Data obtained from such analyses may indicate subclinical and sub-histopathological behavioral, functional, and expected pathological changes. In relation to the cardiovascular system, numerous researchers have attempted to elucidate the participation of the autonomic nervous system in the mechanisms of cardiotoxicity [39].

Currently, the scientific evidence does not give definitive conclusions on the regulation of the sympathetic nervous system in the development of cardiac pathologies. Franchini et al. [40], however, suggested that the increase in neurohormones markers in the sympathetic innervations of the heart may serve as evidence of pathological alterations that can be correlated to early stages of compensated cardiac hypertrophy.

Our findings showed a significant difference between levels of noradrenaline and adrenaline neurohormones, as well as their metabolites, in the cardiac muscle of ketamine-treated rats compared to controls (Figure 5 and Figure 7). These data were suggestive of a decrease in sympathetic activity consistent with the model of depression due to maternal separation (PND12 through PND21), as well as with the group of rats treated with ketamine at doses of 5, 10, and 20 μg/mL. These results were not observed when a single exposure to ketamine was administered.

In the cardiac muscle of experimental animals, we observed a decrease in dopamine levels and its main metabolite, DOPAC, corresponding to the highest dose (20 μg/mL) of ketamine tested (Figure 4). These results were observed in the 21-day group, as well as for the group of rats treated with ketamine at a single dose of 10 mg/kg. These findings corresponded to the three treatment doses and are suggestive of ketamine’s inhibitory activity on cardiac monoamine oxidase enzymes. This inhibitory action favors decreased activity at the synaptic cleft. Although no statistical significance was observed for dopamine levels at doses of 5 μg/mL or 10 μg/mL, the general trend followed a pattern that suggested decreased levels of dopamine and its metabolites compared to controls. Noradrenaline (norepinephrine) and adrenaline (epinephrine) levels were also diminished after ketamine treatment at doses of 5, 10, and 20 μg/mL when compared to control groups. The decrease in cardiac muscle sympathetic response is indicative of a cardioprotective effect of ketamine.

Expanding on the actions of ketamine on cardiac functions, the literature reports that sympathomimetic neural mechanisms do not play stimulating or trophic roles during the progression of cardiac hypertrophy. However, their participation seems to be indirectly linked, which is suggestive of hypertrophied myocardium stimuli towards sympathetic cardiac innervations [41]. It is also known that cellular stores of norepinephrine and acetylcholine are reduced with the progression of heart failure and their depletion generates lower cardiac output in response to sympathetic activation [42].

Physiologically speaking, adrenaline is classified as a hormone, whereas noradrenaline is categorized both as a neurotransmitter and as a hormone, depending on its site of synthesis and secretion [43]. Furthermore, noradrenaline can indirectly stimulate the release of catecholamines from the adrenal medulla. Notably, measurements of the blood concentrations of these neurohormones serve as biomarkers of sympathetic nervous system activation (SNA) [44].

The sympathetic autonomic nervous system (ANS) is influenced by dopamine and dopamine agonists, which in turn generate blood-pressure-lowering effects that are mediated by the inhibition of sympathetic neuronal release of noradrenaline. After stimulation of vascular dopaminergic receptors, mediated by dopamine and L-dopa, these undergo biotransformation into noradrenaline and adrenaline, which exert a positive vasopressor effect, as well as heart-related inotropic and chronotropic effects. Such cardiovascular mechanisms are complex and result from pathways that stimulate vascular and neuronal receptors, including the metabolism of the neurohormones mentioned above and physiological feedback [45].

Another critical neurotransmitter for cardiac assessment is serotonin, or 5-hydroxytryptamine (5-HT). Plasma serotonin is released by platelets and is an important neurohormonal factor with a fundamental role in cardiovascular function [46,47]. Drugs that affect serotonergic receptors are widely used to treat various conditions, such as obesity, depression, psychosis, and migraine. Another category of serotonergic agents includes drugs of abuse, such as amphetamines and methylenedioxymethamphetamine (MDMA) (aka Ecstasy), which can lead to serotonin syndrome that increases cardiovascular risk [48].

Our cardio-chemical findings showed a significant decrease in cardiac muscle 5-HT among the depressed animal groups (PND21) with the three doses of ketamine treatment compared to the control. There was an increase in the turnover ratio of serotonin metabolite to serotonin (5HIAA/5-HT) within the same groups, which suggested that ketamine increased the rate of the metabolism (induction) of cardiac enzymes responsible for this biotransformation. The release or inhibition of 5-HT reuptake or degradation in a chronic form in the peripheral nervous system (PNS), mainly in cardiovascular tissue, exerts direct effects on tissue remodeling, generating cardiac hypertrophy, fibrosis, and valvular degeneration, since those sites have the corresponding receptors [47].

Other observed effects attributed to 5-HT were the regulation of vascular tone, the direct action on cardiomyocytes, and stimulation of the heart’s chemosensitive nerves. Regarding cardiovascular disorders, their occurrences are observed when serotonin signaling is altered or when there is a variation in serotonin concentrations [46]. Evidence shows that in the absence of peripheral serotonin synthesis, blood serotonin is drastically reduced, and such a drop leads to heart failure, concluding that circulating serotonin levels are crucial in maintaining cardiac activity and avoiding cardiovascular disease. The use of serotonin agonists can trigger acute and chronic effects depending on the type and location of receptors. The acute cardiovascular response to 5-HT, called the Bezold-Jarish reflex, leads to intense bradycardia associated with atrioventricular block [49,50,51]. Therefore, lowering 5-HT levels offers new perspectives for its use in treating cardiovascular diseases [46].

Furthermore, the relevance of the serotonergic system to the antidepressant effects of ketamine has recently come to light [52,53]. Studies have shown that the antidepressant properties of ketamine in mice and rats were inhibited by serotonin depletion [53,54,55], suggesting that the serotonergic system is essential for ketamine’s role as an antidepressant [52,53].

Our findings indicated that ketamine in the three doses tested had an important role on the cardiovascular system, contributing to the decrease in serotonin levels in cardiac muscle, as well as an increase in turnover (metabolization) in the same, suggesting that ketamine, when observed from this perspective, can generate protection from cardiovascular hypertrophy.

Expansion of our studies employing molecular research techniques, such as polymer chain reaction (PCR) and OMICS—which are the technologies used to study the function, differences and interaction between various types of molecules that make up the cells of an organism, such as genes, transcripts, proteins and small metabolites—will render more sensitive measurements compared to the histopathological methods. Such studies will provide further evidence of preclinical safety and lack of cardiotoxicity for the employed animal model [14]. We are currently in the process of acquiring funds to proceed with this line of investigation.

## 5. Conclusions

Here we showed that the PPD rat model can be effectively used for the evaluation of candidate drugs’ toxicity and safety.

No histopathological differences were observed between the groups evaluated in the bladder and heart tissues, which shows safety in using ketamine at doses of 5, 10, and 20 mg/kg in the model of rats with PPD.

The technique developed for this study allowed direct measurements of catecholamines and indolamine from cardiac muscle. In addition, direct measurements allowed accurate determinations of local neurohormonal levels, which may be linked to diseases’ processes and progression.

The technique of detection and quantification of neurohormones is unprecedented and shows promise for research and may, in the future, be used to determine a correlation between the concentrations of neurohormones in cardiac tissue and blood plasma. Moreover, such measurements will establish better reference values and expand the use of these neurohormones as markers of heart disease progression.

A limitation of this work was the lack of molecular techniques to better describe the molecular and genetic mechanisms by which ketamine alters the levels of neurohormones in cardiac muscle. The research group is seeking financial support to proceed with this line of investigation.

## Figures and Tables

**Table 1:** Division of the experimental groups.

Group	Description	Treatment Period
**A**	Control (normal, not treated)	
**B**	Control (normal) + saline	PND2 to PND21
**C**	Depressed + saline	PND2 to PND21
**D**	Depressed + ketamine 5 mg/kg	PND2 to PND21
**E**	Depressed + ketamine 10 mg/kg	PND2 to PND21
**F**	Depressed + ketamine 20 mg/kg	PND2 to PND21
**G**	Depressed + Ketamine 10 mg/kg on	Single dose on PND12
